# Fuel Prediction and Reduction in Public Transportation by Sensor Monitoring and Bayesian Networks

**DOI:** 10.3390/s21144733

**Published:** 2021-07-11

**Authors:** Federico Delussu, Faisal Imran, Christian Mattia, Rosa Meo

**Affiliations:** 1 Dipartimento di Informatica, University of Torino, 10149 Turin, Italy; federico.delussu@gmail.com (F.D.); faisalali129@gmail.com (F.I.); 2 Dipartimento di Matematica G. Peano, University of Torino, 10123 Turin, Italy; christian.mattia@edu.unito.it

**Keywords:** bayesian networks, granger causality, hill climbing, brute force, fuel reduction, public transportation, sensors, CAN-bus

## Abstract

We exploit the use of a controller area network (CAN-bus) to monitor sensors on the buses of local public transportation in a big European city. The aim is to advise fleet managers and policymakers on how to reduce fuel consumption so that air pollution is controlled and public services are improved. We deploy heuristic algorithms and exhaustive ones to generate Bayesian networks among the monitored variables. The aim is to describe the relevant relationships between the variables, to discover and confirm the possible cause–effect relationships, to predict the fuel consumption dependent on the contextual conditions of traffic, and to enable an intervention analysis to be conducted on the variables so that our goals are achieved. We propose a validation technique using Bayesian networks based on Granger causality: it relies upon observations of the time series formed by successive values of the variables in time. We use the same method based on Granger causality to rank the Bayesian networks obtained as well. A comparison of the Bayesian networks discovered against the ground truth is proposed in a synthetic data set, specifically generated for this study: the results confirm the validity of the Bayesian networks that agree on most of the existing relationships.

## 1. Introduction

According to the World Health Organization (WHO), air pollution is the second leading cause of noncommunicable diseases, such as stroke, cancer, and heart disease, and of pulmonary diseases, such as chronic obstructive pulmonary diseases and lower respiratory infections. Ambient air pollution accounts for an estimated 4.2 million deaths per year [1]. Around 91% of the world’s population lives in places where air-quality levels exceed WHO limits and the suggested standards for a healthy life [2,3,4]. Air pollution is due to the presence of particulate matter 2.5 (PM2.5), which refers to tiny particles in the air that are two and one-half microns or less in width. Studies suggest that long-term exposure to fine particulate matter may be associated with increased rates of chronic bronchitis, reduced lung function, and increased mortality from lung cancer and heart disease. Furthermore, nitrogen dioxide (NO${}_{2}$) is one of the other main air-quality pollutants of concern and is typically associated with vehicle emissions. The annual EU limit for NO${}_{2}$ was widely exceeded across Europe in 2017. Some 86% of these exceedances were detected at roadside monitoring locations.

**Figure 1:**
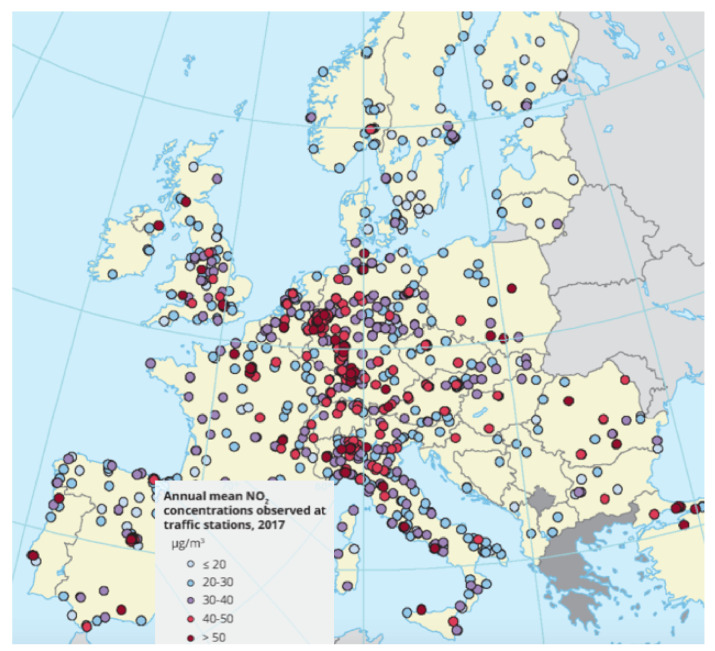
Map of locations with NO${}_{2}$ emissions exceeded over the annual mean limit.

The red and violet colors in the map in Figure 1 show the areas in which the limits were overcome multiple times in past years in European countries. Similar maps are available for the other main air pollutants. In many countries, diseases can only be significantly reduced by improving air quality. Turning air-pollution-reduction goals into policies to combat noncommunicable diseases leads to multiple benefits for the environment, economy, and health. With this work, we address these concerns by putting data science to use at the service of public policies. According to the European Environment Agency, we can reach the goal of a reduction in air pollution by monitoring and modeling air quality, by collecting data using sensors on roads and on vehicles, and by maintaining emission inventories. We should employ emission-control strategies to reduce the amount of private transports; to improve public ones; to reduce their emissions; to increase the use of renewable energy; and to apply contingency measures, new policies, and rules that, for instance, encourage planning of more compact cities.

In this work, we employ machine learning models, specifically, Bayesian networks, to analyze sensor data installed on the buses of a public transport company in a European city. The sensors collect data about the vehicle and its use (acceleration, braking, speed, stop durations with the engine on, etc.) with some contextual information about the vehicle location (such as altitude). An analysis of the sensor data using machine learning algorithms applied using procedures of predictive maintenance can also be used to improve vehicle equipment maintenance, with a reduction in costs due to stop times for faults and repair. Several related works exist in the literature. The application of Bayesian networks for the purposes of monitoring natural resources and applying policies wa proposed in [5]. The majority of the works that monitor fuel consumption in vehicles applied predictive models. Schoen et al. in [6] adopted Artificial Neural Networks (ANN) to predict average fuel consumption in a fleet of heavy vehicles. They adopted a data summarization technique of the consumption based on distance rather than time in order to eliminate a conversion of the scale for the prediction of average fuel consumption. We also apply a similar technique in this work because we build models that employ the fuel consumed per kilometer. Perrotta et al. [7] compared multiple machine learning models—support vector regression (SVR), random forest (RF), and artificial neural networks (ANN)—to predict fuel consumption in heavy vehicles. Moradi et al. [8] used multiple models in cascade and confirmed that ANN outperforms the other models. The goals of these works were to reduce costs and to obtain better routing of the fleets even though they found it difficult to determine an accurate estimation of the fuel level. Yao et al. in [9] used smartphones to collect vehicle mobility data based on their global positioning system (GPS) combined with data from on-board diagnostics (OBD) terminals to predict fuel consumption based on taxi-drivers’ driving styles. They compared ANN, SVR, and RF and showed that all of them reach satisfactory prediction performances. Random forest achieved a superior accuracy. Rimpas et al. in [10] selected some parameters for monitoring vehicles retrieved through the OBD-II diagnostics protocol and related them to vehicle operation and fuel consumption. They collected the proportion of oxygen in exhaust gases using a Lambda Sensor and adjusted the fuel quantity measured by a short-term fuel trim (STFT) sensor related to the immediate change in fuel flow and used as a proxy of the accelerator pedal pressed by the driver. They collected the air flow as measured by a mass air flow sensor (MAF) as a measure of engine malfunction, a vehicle speed sensor (VSS), and the value of the engine coolant temperature (ECT) sensor where the coolant temperature affects engine overheating and fuel consumption. The authors in [11] quantified the uncertainty in measuring fuel consumption, both in light and heavy vehicles. They show that, in urban conditions, the uncertainty reaches 7%. In [12], the authors considered the prediction of fuel consumption in public buses using a multivariate data set including several explanatory variables. They compared RF, gradient boosting (GB), and ANN. Based on their analysis, RF produces a more accurate prediction compared to both GB and ANN. In [13], the authors included weather variables for the task of fuel prediction and considered them useful for an accurate prediction. Quite often in the above studies, the sample vehicles (in terms of make, model, and age) were comparable so that the type and status of the vehicle does not influence fuel consumption. We made a similar choice in the selection of heavy vehicles (buses of the same model, type, mass, length, and age).

In this work, we used sensors with the sole purpose of collecting data about fuel consumption and monitoring the drivers’ usage of the bus’s resources (fuel, breaks, acceleration, and air conditioning). The goal was to monitor fuel consumption and its contextual conditions with the ultimate objective to provide a descriptive and explainable model of the variables that influence and cause fuel consumption and that ultimately produce air pollution. We employed Bayesian networks that permit us to afford a unique model with multiple tasks: description with a graph of the dependence relationships between the variables, identification of the variables that are independent from the target, selection of the variables that have an impact on the target, quantification of the amount of impact on the target, prediction of the target, simulation of the variables in a scenario, and intervention in the scenario by changing some of the variables.

The first contribution of this work is to provide a public data set [14] on sensors installed on board public transports with information about vehicle usage and fuel consumption. Sensors communicate their measures via the controller area network (CAN-bus), a specialized internal communications network that interconnects components inside a vehicle [15]. CAN is a robust vehicle standard designed to allow micro-controllers and devices to communicate with each other’s applications without a host computer. It is a message-based protocol, originally designed for multiplex electrical wiring within automobiles, but it can be applied to many other contexts. For each device (sensor and actuator), the data in a frame are transmitted sequentially. Thanks to this, the vehicle turns out to be an advanced, computerized control system available on board and capable of sensor data storage.

Thanks to the collected data, we assessed the sensor outcomes to support decision making. We employed Bayesian networks (BN) as an essential tool that is able to provide descriptive and explainable models of the relationships between the monitored variables, and dependence relations that might also represent the cause–effect relationships [16]. In fact, BN captures the independence and the conditional independence among the variables: in a BN, we represent variables with nodes and dependence relationships with edges. The presence of a path connecting a variable *V* with a target variable *T* makes it clear that we should change the values of *V* in order to modify the values of the target (query) variable *T*. Instead, a change in variables not connected within a path including the target should not cause any effect on it. The main contribution of this work is to provide a BN on the variables monitored by sensors connected in CAN-bus. These BN show which variables we should change to control the fuel consumption variable. Furthermore, BN also supports simulation of the behavior of the system. We use this feature of the BN model because we generate synthetic data of a set of sensors that obey a known ground truth [14]. The purpose is to verify correspondences between the cause–effect dependencies reconstructed from the data and the true ones. We made these synthetic data publicly available too [14].

BN is employed also to perform an assessment of the observed phenomena and to perform an intervention analysis on the causal variables so that the monitored target can be improved. As a result, we can provide the results and suggestions to drivers and policy-makers with the goal of improving air quality and reducing costs for fuel. This is the third contribution of this work. One of the main results of this intervention analysis is to show that a change in the vehicle paths (longer but with a reduced slope) turns into a decrease in fuel consumption. Other results concern the quantification of the impact on fuel consumption of air conditioning and of brake usage. 

The main difficulty with BN is that the search space of the possible alternative models increases in a super-exponential way in the number of variables (graph nodes) [17]. Therefore, it is customary to employ approximate algorithms [18,19,20] driven by heuristics that are used to rank and evaluate the alternatives. The results are that the algorithms might converge to different and suboptimal solutions but in tractable times. Their results, as we experienced and show in this work, might differ. In this paper, we deal with some representative algorithms for BN synthesis from data that are popular in the BN community [19,20]. We use the BIC score [21], a derivation of the likelihood of the data under the assumed BN model, as a heuristic to evaluate the alternative networks. We revised them and compared their solutions on the sensor data by providing a brute force alternative. Brute force converges to the global optimum of the BIC score within the search space. The brute force alternative is possible (provided the number of variables is kept limited to some units) thanks to the opportunity that high-performance computing gives us. It makes the workload efficient by distributing the computation among multiple servers and CPUs, and their execution in parallel. This is the fourth contribution of this work and one of the novelties of our approach: a comparison of the results of different algorithms for BN generation from data that allows us to rank them and to evaluate how closely they reach the overall optimum of brute force. This is not so common in the BN community, since BNs are usually initially provided by domain experts and later validated against evidence from data [22,23]. To overcome the discrepancies among BNs, we compared and ranked them by proposing and adopting an alternative method: Granger causality [24]. This is one novelty of our approach and the last, but not least, contribution of our work. Granger causality and its statistical test employ vector auto-regression (VAR) as a tool to predict the target in time with the aid of multiple variables (the variables that are in the pathway from causes to the effect). In its essence, the statistical test in Granger causality method verifies that the prediction of the target, with the aid of the cause variables, is better than without them. The application of this latter criterion is possible only when the flow of values of these variables is stored in time. Granger causality is commonly judged as a weaker principle than the stricter principle of probabilistic dependency between cause and effect. With Granger causality, the existence of a causality relation between cause and the effect is verified only in time thanks to the ability of the cause to predict and anticipate the effect in time [25,26].

## 2. Materials and Methods

### 2.1. Sensor Data

The data set was collected by sensors on a fleet of bus vehicles. The data set contains records for a fleet of 24 vehicles over 43 dates comprising dates between January and August 2019. It is publicly available at [14]. Sensors from the on-board diagnostics (OBD) interface collects kinematic variables such as speed, acceleration, engine speed (RPM), load (mass), and road grade. For each vehicle, the sensors perform measurements during a path from departure to arrival bus-stop; thus, the data are not sampled regularly according to time. We have a collection of multiple path records for each date on which the vehicle is driven. (For each vehicle and date, the number of records generally comprises between 100 and 400 units). The variables measured during the path include the physical properties of the travel (path length, duration, and change of height), time variables (time intervals spent coasting, braking, or in motion), and the fuel consumption of the vehicle during this time intervals. Unfortunately, we could not include the weather condition and the road type of the tracks, even if we assumed that, in the domain of public transportation, the road type is almost always metropolitan.

Our work aims to apply Bayes networks and Granger causality to study the causal association between variables, especially on fuel consumption.

### 2.2. Feature Selection and Construction

We constructed a set of representative features over which we performed our experiments.

- Original feature collection can be grouped as follows:
  - Path variables
    ∗ HDIFF (m): difference in altitude between departure and arrival bus-stop
    ∗ DIST (m): distance covered during the travel
    ∗ MASS (kg): mass of vehicle and passengers
  - Time Interval variables (s)
    ∗ TMTOT: total time of the travel
    ∗ TMAIR: time with air-conditioning on
    ∗ TMCOAST: time spent coasting
    ∗ TMBRAKE: time spent using the brakes
    ∗ TMMOTION: time spent with vehicle in motion
      From this variables we can derive:
      ∙ TMTRACTION: TMMOTION − TMCOAST − TMBRAKE: time spent in traction, that is, pressing the accelerator pedal
      ∙ TMSTOP: TMTOT − TMMOTION: time spent with the stopped vehicle with the engine on
  - Fuel consumption variables (mL)
    ∗ FUELSTOP: fuel consumption in TMSTOP time
    ∗ FUELMOTION: fuel consumption in TMMOTION time
- New features collection is contructed as follows:
  - avg_slope (%): HDIFF/DIST
  - mass (ton): MASS/1000
  - brake_usage (%): (TMBRAKE − TMCOAST)/TMTOT
  - air_cond_ptime (%): TMAIR/TMTOT
  - stop_ptime (%): TMSTOP/TMTOT
  - fuel_per_km (L/km): (FUELSTOP + FUELMOTION)/DIST
  - accel (m/s^2^): 2 × DIST/(TMMOTION × TMTRACTION)

Concerning the derived variables, we can state the following:

- In the data set, buses travel at all different lengths and durations. We chose to divide the total fuel consumption by distance to perform a better comparison among buses traveling at different lengths. For the same reason, we decided to normalize all of the time variables involved in the analysis so that they represent a fraction of the total travel time;
- The variable *brake_usage* was created as an indicator of the good practice of choosing coasting instead of braking. This variable is negative when the time spent coasting is greater than the time spent braking, zero when these fractions are equal, and positive otherwise;
- The variable *stop_ptime* includes only the idling time, that is, the time spent with the vehicle not in motion but with the engine on;
- The variable *accel* is obtained as the result of a simplified model of bus travel. We assume that the bus travels starting, at time 0 with $v_{0}=0$. We assume that the velocity increases linearly with a constant acceleration (that is presumed to be an important variable for the prediction of fuel consumption) until the time is equal to TMTRACTION. Then, we assume that the bus velocity starts to decrease linearly for a time equal to the sum of time spent coasting and braking, so that when time is equal to TMMOTION (i.e., TMTRACTION + TMBRAKE + TMCOAST), the final velocity turns out to again be null: $v_{f}=0$. The *accel* value can be easily derived in this simplified model by observing that the length of the travel is equal to the area of the velocity graph in a velocity–time diagram or, more formally, by solving
  $$\left\{\begin{array}{c} s=\frac{1}{2}a_{1}{t_{1}}^{2}+v_{1}\left(t_{2}-t_{1}\right)+\frac{1}{2}a_{2}{\left(t_{2}-t_{1}\right)}^{2}\\ v_{1}=a_{1}t_{1}\\ 0=v_{1}+a_{2}\left(t_{2}-t_{1}\right) \end{array}\right.$$
  where *s* is the travelled distance, $t_{1}$ = TMTRACTION, $t_{2}$ = TMMOTION, $v_{1}$ is the velocity at time $t_{1}$, $a_{1}$ is the positive acceleration we are looking for, and $a_{2}$ is a negative acceleration (not involved in fuel consumption).

We show the main statistics (mean, standard deviation, and min–max range) of the new feature collection for the data set on which we perform our experiments in Table 1. We can observe that the vehicles mass is around 20 tons, that the path is generally on a plain ground (from the mean *avg_slope*), and that the fuel consumption is around 0.6 L per km.

### 2.3. Algorithms for Bayesian Network Learning

In this section, we outline some of the algorithms to learn causal models from the observed data. Learning a Bayesian network occurs in two steps: *structure learning* and *parameter learning*. Suppose that learning a BN with DAG G and parameters Θ from a data set D having *n* observations is driven by the following:

P(G,Θ∣D)=P(G∣D)·P(Θ∣G,D)

Structure learning is involved in learning P(G|D): it aims to find the DAG G that incorporates the dependence structure between the variables of the data D. In contrast, parameter learning is focused on P(Θ|G,D) and consists of estimating the parameters Θ given G. Suppose that the parameters are independent in distributions; then, they can be learned in parallel for each node $X_{i}$ as follows:

$$P\left(\Theta |G,D\right)=\prod_{i=1}^{N}P\left(\Theta_{X_{i}}|Pa_{i},D\right)$$

where, with $Pa_{i}$, we represent the set of parent nodes of $X_{i}$ (connected with a directed edge, incoming in $X_{i}$) and, with $\Theta_{X_{i}}$, we represent the set of parameters of the conditional distribution of $X_{i}$ given its parents $Pa_{i}$ in G. Learning the structure of BN is an NP-hard problem and computationally challenging. Suppose that there are *N* nodes; then, the possible arcs are N(N−1)/2 and the number of DAGs grow super-exponentially as the number of nodes *N* increases. Hence, only a small number of the possible alternative DAGs can be explored in a reasonable time. There are three main possible approaches used in the structure learning of the BN: *score-based, constraint-based, and hybrid*. Each based on a different statistical criterion.

- **Score-based approach** is a general class of optimization techniques to learn BN structure. Each learned BN is assigned a network score based on its *Goodness-of-Fit*; the algorithm then tries to maximize the network score. Score-based approach examples include *simulated annealing, greedy search* [27], *genetic algorithms* [28], and *hill climbing* (HC) [19].
- **Constraint-based approach** first identifies pairs of nodes $\left(X_{i},X_{j}\right)$ that are connected with an arc, regardless of its orientation. These nodes cannot be separated by other subsets of nodes; this is tested heuristically using a conditional independence test. The algorithm then distinguishes the v-structure among all of the pairs of non-adjacent nodes $X_{i}$ and $X_{l}$ with a common neighbor $X_{j}$ using the separating sets found earlier and sets the remaining arc directions using the rules from Chickering [20] to obtain CPDAG (completed partially directed acyclic graph). Some examples include *Grow-Shrink* [29] and *Interleaved Incremental Association* (Inter-IAMB) [30].
- **Hybrid approaches** are constraint-based and use restriction to reduce the candidate space of DAGs; they are score-based and use *maximize* implementations to find the optimal DAG in the restricted space by implementing any combination of constraint-based and score-based algorithms. Hybrid approaches include *Max-Min Hill Climbing algorithm* (MMHC) [18], *Restricted Maximization* (RSMAX2) [31], and *Hybrid HPC* (H2PC) [32].

#### 2.3.1. Hill Climbing Algorithm

The hill climbing algorithm belongs to the class of greedy search algorithms. Hill climbing (HC) assigns a network score (*Goodness-of-Fit*) to the candidate BNs, and heuristic algorithms strive to maximize the network score, since a higher value means a better fit. HC starts from a DAG structure, and then it adds, reverses, and deletes arcs until the network score no longer improves [19]. The network score can be the *Bayesian Information Criterion* [33] (BIC) or *Akaike Information Criterion* [34] (AIC) for both discrete and continuous data sets.

#### 2.3.2. Restrictive Maximization Algorithm

The restrictive maximization algorithm belongs to the class of hybrid approaches. RM achieves faster structure learning by restricting the search space and by implementing a combination of constraint-based and score-based algorithms [31].

#### 2.3.3. Brute Force Algorithm

In this work, we introduce the brute force algorithm to afford the computational complexity of complete exploration of the search space of the possible BN alternatives. We take advantage of the parallel computing technology provided by HPC4AI (Turin’s High-Performance Centre for Artificial Intelligence https://hpc4ai.it/, accessed on 8 July 2021). The brute force formalization and implementation is one of the original contributions of this work. We split up the search space for model selection and assign each to an independent processor that delivers the best BN of the corresponding subspace. Finally, these results are compared to choose the very best model. Each candidate BN is assigned with a network score “*Goodness-of-fit*”. The brute force algorithm returns a BN with the maximum score since a higher score means a better fit. We used a score derived from the Bayesian information criterion (BIC) as implemented in the R Library [35]; this network score is suitable for both continuous and discrete data sets.

The idea of the brute force algorithm is to partition the space for all possible Bayesian networks and to allocate each partition to a different processor, such that each processor in parallel executes the task to evaluate the BIC score of all networks in its partition. Each network is represented as a vector—a binary configuration of as many bits as the possible arcs in the networks. Each bit in the vector represents whether the corresponding arc is present or absent in the network.

The algorithm starts with an input data set D containing *N* variables. AllArcs is a matrix (p × 2) with p=N(N−1)/2 being the number of possible (undirected) arcs. Each row in the matrix represents an arc (from–to): the first column represents the starting node, and the second represents the ending node. Each pair of nodes is identified by a matrix row index from 1 to p.

$k_{1}<$ p is the number of arcs that are actively considered by each processor, and the processor is free to vary in anyway in combination with the remaining arcs that instead are fixed. The different processors have a different configuration in terms of present/absent arcs that are fixed in the remaining subset of $p-k_{1}$ arcs. FixedArcsPresence is a vector of length $p-k_{1}$ containing information related to the arcs for which the presence/absence is fixed for that processor. $p-k_{1}$ is the prearranged arcs (or pairs of nodes). In total, we have $2^{p-k_{1}}$ available processors. Each processor runs the brute force Algorithm 1, with FixedArcsPresence as an input argument. FixedArcsPresence is a vector of the ordered list representing the presence/absence of each of the prearranged $p-k_{1}$ arcs. Each element in this vector corresponds to a different nodes pair with values 0,1 such that FixedArcsPresence[i]=1 if the *i*th pair of nodes is considered by that processor to be connected; otherwise, it is 0. The processors are executed in parallel, where each processor has a different realization of FixedArcsPresence. For each total configuration of arcs present or absent, from the fixed part and the variable part, the processor evaluates the BIC score of the corresponding Bayesian network with the goal of finding the one with the maximum value. Regarding the determination of the arcs’ directions, defined within the algorithm, it establishes whether each arc is oriented according to the direction taken as the reference in such a matrix or the other way around and evaluates the BIC score for both arc directions. At the end, the maximum score among the scores found by the processors is selected and so is the corresponding Bayesian network.

However, some care should be taken when Bayesian networks are learnt from the data. It should be kept in mind that networks learned from observational data may establish some relationships that are hard to explain based on our prior knowledge of the domain. Some relationships may reveal aspects of phenomena that we did not expect, some may be explained by introducing exogenous variables acting as confounders, and the influence of variable $X_{i}$ on another variable $X_{j}$ may be mediated by an unobserved variable $X_{l}$ that is not included either in the model or in the available data. Moreover, we should take into account that the model, due to a lack of flexibility, could be unable to accurately describe the phenomenon. For example, the assumption of Gaussianity may be inadequate for our data, and adapting the variables to multinomial assumption through discretization may lead to mutual information loss.

Therefore, we cannot expect to find a rational justification for each connection, but we can apply critical thinking to extract helpful insights based on what the data supports.

### 2.4. Granger Causality

We performed a model evaluation of the Bayesian networks employing the statistical concept of Granger causality that applies to the time-series domain [24]. In the following, we provide a formalization of the application of the concept of Granger causality to the evaluation of Bayesian networks. Later, in Section 3.2, we apply this method to the task of ranking and comparing Bayesian networks, resulting from the application to the same data of different, approximate, and heuristic-driven algorithms. The provided solution can solve a data analyst’s uncertainty for the choice among them. These concepts, to the best of our knowledge, are original in their application to the validation of Bayesian networks and in its formalization.
**Algorithm 1** Brute force algorithm for learning the best Bayesian network structure.
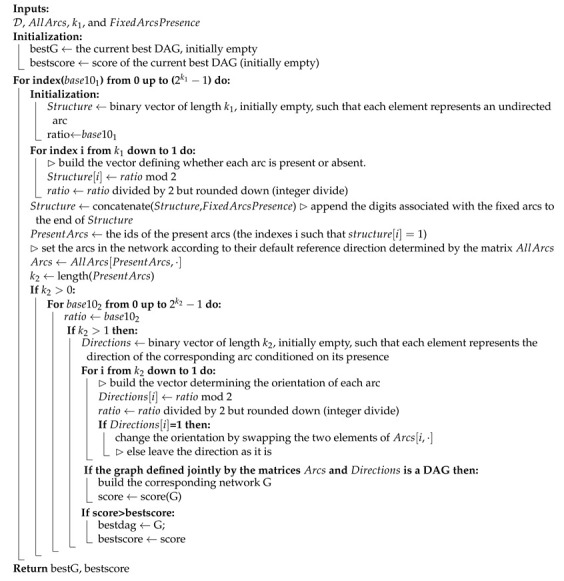


#### Granger Test

Given a stationarized  multivariate time series $t^{\left(l\right)}$, including variables *A* and *B*, we want to establish if $A\overset{\left(g\right)}{\Rightarrow }B$. (The time series must satisfy the stationarity condition that is assessed for each feature by the Augmented Dickey Fuller test (ADF) with significance level 0.05. If the $S_{i}$ feature gives rise to a time series that is not stationary, we iteratively apply first-differencing $S_{i}\left(t\right)\to \left[S_{i}\left(t\right)-S_{i}\left(t-1\right)\right]$ and repeat ADF until we reach stationarity). Notation $A\overset{\left(g\right)}{\Rightarrow }B$ denotes that *A* Granger-causes *B*. We performed a Granger test by comparing the two auto-regressive models:

(1) $$\begin{array}{cc} \;\;\;B_{t} & =\sum_{l=1}^{q}\beta_{l}B_{t-l}+\epsilon_{t} \end{array}$$

(2) $$\begin{array}{cc} \;\;\;B_{t} & =\sum_{l=1}^{q}\beta_{l}B_{t-l}+\sum_{l=1}^{q}\alpha_{l}A_{t-l}+\epsilon_{t} \end{array}$$

The Granger test is an *F*-test with null hypothesis $H_{0}:=\left\{\alpha_{l}=0;\;l=1,\ldots ,q\right\}$. The success of the test implies that $A\overset{\left(g\right)}{\Rightarrow }B$, that is, *A* has a predictive power on *B* since its lag coefficients $\alpha_{l}$ in the second auto regressive model are significantly different from 0. For our multivariate time series $t^{\left(l\right)}$, we conducted a Granger test for each possible ordered distinct pair from feature set $\left\{S_{1},S_{2},\ldots ,S_{N}\right\}$. Here, we denote the variables with symbols $S_{i}$ because we want to highlight that each gives rise in a vehicle to a set of time series (one for each vehicle in time). We stored the result of the $t^{\left(l\right)}$ tests in a Granger matrix $G^{\left(l\right)}$:

(3) $$G_{i,j}^{\left(l\right)}=\left\{\begin{array}{c} 1:S_{i}\overset{\left(g\right)}{\Rightarrow }S_{j}\;\mathrm{for}\;t^{\left(l\right)}\\ 0:else \end{array}\right.$$

We fit a vector auto regressive model (VAR) over the time series $t^{\left(l\right)}$, and the maximum-lag order *q* was selected automatically according to the AIC criteria. We then performed a Granger test for each pair $\left(S_{i},S_{j}\right)$. The related F-test was performed with a significance level of 0.1. We made this choice because we observed that, with a level of 0.05, we have an average decrease of 5% in the rate of success of the Granger test over the set of time series of the experiments.

We frame our data set as a collection of multivariate time series $\left\{t^{\left(l\right)};\;l=1,\ldots ,T\right\}$. We performed the Granger test for each time series $t^{\left(l\right)}$ and for each ordered variable pair $\left(S_{i},S_{j}\right)$. The whole test results were then stored as a collection of Granger matrices $\left\{G^{\left(l\right)};\;l=1,\ldots ,T\right\}$. This collection is successively used for validating the Bayes networks.

## 3. Results

### 3.1. The Discovered Bayes Networks

We illustrate the Bayes networks discovered from the algorithms introduced in Section 2.3 applied on the data set with the constructed features described in Section 2.2. We initially conduct an analysis on the found relationships between data set features based on our knowledge about the data set domain: public transportation. We then introduce the diverse applications of Bayes networks such as feature selection for a supervised prediction task and intervention analysis in order to perform decision making.

#### 3.1.1. Bayesian Networks Analysis

We name the discovered Bayesian networks after the algorithms introduced in Section 2.3 as employed for their construction:

- HC: Hill climbing
- RM: Restrictive Maximization
- BF: Brute Force

We group the collection of links found by the networks in Table 2 as follows:

- Common Links (CL)
  We have 10 links on which the three networks agree both on presence and direction, identifying reasonable dependencies. Specifically, we have that *brake_usage* and *accel* are both caused by *avg_slope* and *stop_ptime*: this can be interpreted with the fact that a steep path in which we have to slow down or stop, if necessary, implies more frequent use of the brakes or, conversely, in order to ride up a steep path, to use the accelerator pedal. *fuel_per_km* is caused by variables *avg_slope*, since a steeper path causes a greater consumption; *stop_ptime*, since the fuel consumption of a stopped vehicle and the engine still on is higher; *brake_usage*, since more frequent use of the brakes is related to a greater *stop_ptime*; *mass*, since a heavier vehicle (full of passengers) requires greater fuel consumption; and *aircond_ptime*, since air-conditioning is expensive in terms of fuel. Moreover, we have that *aircond_ptime* is caused by *mass*; this can be explained by the fact that a higher mass implies a greater number of persons, which increases the temperature within the vehicle and requires the use of air-conditioning.
- Common Links with Discordant Direction (CLDD)
  We have three variable pairs on which the three networks agree on the link presence but are discordant on the direction. We discuss the relationship for each of the three related variable pairs.
  For *accel* and *brake_usage*, we think that more frequent use of the brakes implies subsequent use of the accelerator: according to this, *brake_usage* causes *accel*, as stated by HC and RM. BF states the opposite, and it seems reasonable that the use of the accelerator may lead to successive use of the brakes for decreasing the speed. We cannot infer the actual direction of the causal relationship between the two variables without auxiliary information concerning the traffic condition and driving behavior. Unfortunately, the data set does not contain these features. We only have a proxy of these conditions from *stop_ptime* and *brake_usage*.
  For *accel* and *fuel_per_km*, we retain that more frequent use of the accelerator causes a higher consumption, so *accel* causes *fuel_per_km*, as stated by HC and BF (while RM states the opposite).
  For *mass* and *brake_usage*, we retain that a higher mass of the vehicle implies a higher probability that someone on the bus requires leaving the vehicle, which follows the requirement that the bus driver needs to brake and to: so *mass* causes *brake_usage*, as stated by HC and RM (while BF states the opposite).
- Uncommon Links (UL)
  We have four links for which the three networks do not agree, both on presence and direction.
  For the variable pair *mass* and *accel*, we think that a heavier vehicle requires more frequent use of the accelerator to reach its destination, so *mass* causes *accel*, as stated by HC, while BF states the opposite and RM does not find a relationship.
  The causal relationship between *mass* and *stop_ptime* is found only by HC. It appears reasonable and in agreement with the fact that *mass* causes *brake_usage*.
  The causal relationship between *slope* and *mass* is found only by BF. This link is more difficult to interpret; maybe the dependence between slope and mass may be explained by the fact that a low value of slope may be a proxy for identifying a crowded region of the town, where more people get on the bus and therefore the mass increases.

From our considerations, we observe that the links found by the networks can be explained with arguments concerning the domain of public transportation. Moreover, we notice that, for links with a discordant network direction (CLDD), a feedback link over a variable pair may exist though it is not contemplated by the DAG structure found by the Bayes network algorithms. That is, given a variable pair and a network construction algorithm, we find a directed causal relationship that may not be the only one in the considered domain. For example, we are uncertain on the causal direction for the pair (*brake_usage* and *accel*). Indeed, excessive acceleration may lead to the use of the brakes and the use of brakes ensures the later use of the accelerator during the same bus path. Therefore, the true relationship between this pair may be a feedback link (a cycle). Unfortunately, we know that the network construction algorithm excludes the formation of loops and it will never be found.

**Table 2:** Link collection found by the three Bayes networks: hill climbing (HC), restrictive maximization (RM), and brute force (BF). The collection is grouped as common links (CL), common links with discordant direction (CLDD), and uncommon Links (UL). For CLDD and UL, we specify the networks for which the links are present.

	CL	CLDD	UL
1	avg_slope → brake_usage	accel → brake_usage (BF)	mass → accel (HC)
2	stop_ptime → brake_usage	brake_usage → accel (HC, RM)	mass → stop (HC)
3	avg_slope → accel	brake_usage → mass (BF)	accel → mass (BF)
4	stop_ptime → accel	mass → brake_usage (HC, RM)	slope → mass (BF)
5	avg_slope → fuel_per_km	fuel_per_km → accel (RM)	
6	stop_ptime → fuel_per_km	accel → fuel_per_km (HC, BF)	
7	brake_usage → fuel_per_km		
8	mass → fuel_per_km		
9	aircond_ptime → fuel_per_km		
10	mass → aircond_ptime		

These observations highlight the difficulty of Bayes networks in determining the causal direction between variables, which may be chosen after network construction with the aid of the expert knowledge.

Concerning fuel consumption, we observe that all of the networks agree on the causal relationship of the variables (*brake_usage*, *avg_slope*, *air_cond_ptime*, *stop_ptime*, and *mass*) over *fuel_per_km* while the reasonable relationship of *accel* causing *fuel_per_km* is found by HC and BF. RM states the opposite relationship, which we consider inexact.

**Figure 2:**
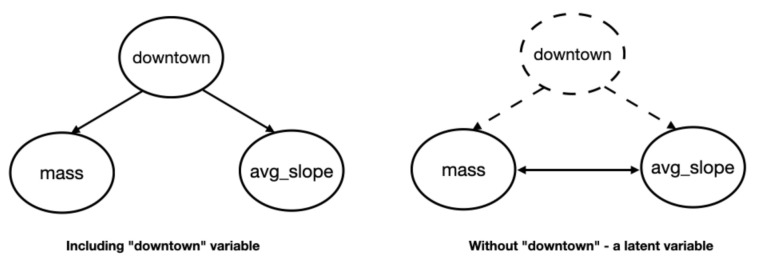
Detail of the uncertainty in Bayesian networks due to the presence of a latent variable.

In Figure 2, we show the details of the BN that might have been extracted if the presence of a latent variable on the typology of the location (e.g., downtown) were not left as a latent information but were explicit. As a consequence of the presence of an unobserved variable, which is a common cause of other variables (*mass* and *avg_slope*), we observe the situation on the right, with a possible mutual link between the effects that are not easily explained alone. This common situation is recognized also in the literature [36], and the BN are deemed as equivalent from the viewpoint of the algorithms (but not by the experts).

#### 3.1.2. Feature Selection and Target Prediction with Bayesian Networks

**Figure 3:**
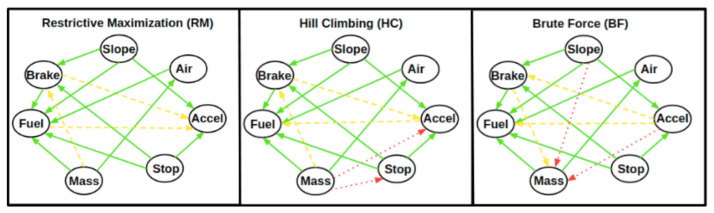
Bayes networks discovered by the algorithms (RM, HC, and BF) over the feature set *accel* (Accel), *avg_slope* (Slope), *air_cond_ptime* (Air), *brake_usage* (Brake), *mass* (Mass), *fuel_per_km* (Fuel), and *stop_ptime* (Stop). The set is described in Section 2.2. The green continuous arrows are common links (CL) between the networks, the yellow dashed arrows are common links with discordant directions (CLDD), and the red dotted arrows are uncommon links (UL), as presented in Table 2.

The Bayesian networks can be applied to perform feature selection for a given supervised prediction task; we considered multivariate linear regression of a given feature node $x_{v}$, where we say $x_{v}$ is the *target*. Given the feature set $X=\{x_{1},x_{2},\ldots ,x_{N}\}$, we define $X_{-v}=X\setminus \left\{x_{v}\right\}$ and inquire which features of $X_{-v}$ should be selected to perform a regression on *target* $x_{v}$. Given a Bayes network B, we introduce the feature set $P_{v}^{\left(B\right)}$ as the set of parent nodes of $x_{v}$ with respect to B (as an example, from Figure 3, we have for the Brute Force network (BF) that the parent set of brake node is $P_{\mathrm{brake}}^{\left(BF\right)}=\left\{\mathrm{accel},\mathrm{stop}\_\mathrm{ptime},\mathrm{avg}\_\mathrm{slope}\right\}$).

We performed feature selection by choosing the features of parent set $P_{v}^{\left(B\right)}$ for the regression of *target* $x_{v}$. We notice that this feature selection is feasible only when $P_{v}^{\left(B\right)}\neq \emptyset$. For example, we observe from Figure 3 that *avg_slope* does not admit a non-null parent set for any of the discovered Bayes networks.

We evaluated the performance of the target prediction using the regression model constructed with the parent feature selection. In the evaluation of the performance, we applied the 10-fold cross-validation score on the root mean squared error (*rmse*) of the regression. We then compared the 10-fold averaged *rmse* with the mean and standard deviation of the *target* feature, which can be obtained from Table 1. We report the *rmse* performance for each feature and for each parent set identified by our Bayes network collection in Table 3.

We observe that, for each *target* feature, the CV scores tend to be of the same order of magnitude with respect to the *target* standard deviation and are generally smaller. Therefore, we can state that the target regression with respect to the parent set tends to provide reasonably low discrepancy errors. When it is possible, we can employ multiple CV scores in order to compare the Bayes networks in order to assess their ability to perform feature selection by identifying different parent sets. To take an example, if we consider target *fuel_per_km*, the networks HC and BF have better performances with respect to RM in terms of parent feature selection for regression. In fact, HC and BF identify a parent set made by all of the features $X_{-\mathrm{fuel}\_\mathrm{per}\_\mathrm{km}}$ while the RM parent set does not include the *accel* feature.

In order to perform a more comprehensive study on feature selection, we compared parent set selection with the variance inflation factor (VIF) technique. VIF is a feature-selection technique [37] that has the goal of reducing multicollinearity in a multivariate data set given the feature set $S=(s_{1},s_{2},\ldots ,s_{d})$.

We compute the variance inflation factors collection $S^{\left(\mathrm{VIF}\right)}=\left(V_{1},V_{2},\ldots ,V_{d}\right)$. We evaluate for each feature $s_{l}$ the quantity $R_{-l}^{2}$, that is the R-squared of regression of feature $s_{l}$ with respect to $S_{-l}$ and the corresponding variance inflation factor $V_{l}=1/\left(1-R_{-l}^{2}\right)$. We remove the feature $s_{m}$ with the highest inflation factor if $V_{m}>5$. (For high $V_{m}$ we have that feature $s_{m}$ has high collinearity with respect to the other features and has a scarce impact in the regression). We then repeat iteratively the same procedure by recomputing the VIF and removing one feature at each step of the iteration until we reach $V_{m}<5$. We apply VIF on $X_{-v}$ for feature target $x_{v}$ but we are not able to perform feature removal since the VIF feature values are of order $10^{-2}$ or less, suggesting that our data set does not exhibit multicollinearity. Therefore we can reasonably use the Bayes networks as an alternative valid instrument to perform feature parent selection.

#### 3.1.3. Intervention Analysis

One interesting feature of Bayesian networks is the possibility to estimate the impact of the intervention on variables using just observational data. This is an advantage because we do not need to perform costly and, in some cases, impossible experiments. We say we perform an intervention on a variable when we treat it as fixed for the whole data set. The goal of this task is to estimate the impact on the target of the action of control and to change the values on one of its causes. This is an original and valuable contribution of our work since this intervention aims to reduce fuel consumption and provides actionable knowledge as a result of sensor data analysis.

To estimate the impact of intervention without using the experimental data, we follow the approach provided in [22,38]. For a Gaussian BN, the causal effect of *X* on *Y* is determined as follows:

- We determine the set of parents of *X* in the BN graph (we denote it as Pa(X)); it is the set of variables directly connected to *X* in the graph.
- We perform a linear regression of *Y* on *X* and Pa(X); it computes the target as a function of the other variables on which it depends; and
- The coefficient of *X* provides us with the causal effect of *X* on *Y*: each coefficient quantifies the amount of impact of each cause to the target.

Assuming the BN structure obtained using the brute force algorithm to be true and restricting our attention to fuel_per_km as the target variable, we obtain the variables that have an effect on the target. They are {slope,mass,air_cond_ptime,stop_ptime,brake_usage, and accel}. Table 4 shows them together with the other variables (the adjustment set). In the determination of the contribution of each single cause to the effect, we need to maintain the values of the adjustment set in order to block-out their causal effect on the target and concentrate only on a single cause (adjustment criterion) [39]. All of these variables are included as inputs in the regression for the determination of the target; later, we consider the variation in the target as a function only of a single causal variable for the quantification of its impact on the target.

Table 4 summarizes all of the possible impacts that the variables have on the target fuel_per_km. This is exactly the added value of Bayesian networks compared to the usual analytical studies based on prediction models: we can forget about the impact on the target of the remaining variables that are not directly connected to the target because they cannot have a direct impact on it. The (causal) variables of the target are directly connected to it and are exactly those ones that can have an effect on it. This effect is precisely quantified by the amount called “causal effect”: it measures the increase in the target for any unit of increase in the corresponding causal variable.

Using the values in Table 4, we can consider the following about the driving styles that are suitable to reduce fuel consumption:

- If we decrease of one unit air_cond_ptime, we can expect a decrease of 0.107 units in fuel_per_km;
- If we decrease of one unit brake_usage, we can expect a decrease of 0.206 units in fuel_per_km; and
- We can obtain similar considerations about mass, obtaining a decrease in fuel_per_km of 0.012 for each decrease in a ton of mass.

We observe that the causal effect for *avg_slope* is relatively high with respect to the other variables. This can be explained by the fact that, for the considered angle interval $\left(0^{\circ },10^{\circ }\right)$, the corresponding slope, that is the tangent of the angle, ranges in the interval (0,0.18). Then, the corresponding slope variations are of the order $10^{-1}–10^{-2}$. Therefore, since the maximum slope variation is 0.18, that is, very small with respect to the unit value, we have that the corresponding maximum *fuel_per_km* variation is comparable to 6.635×0.18=1.19 L/km. This latter variation is compliant with the domain knowledge. Although the mass and avg_slope variables are not under the control of the driver, this information can still be useful. A decision-maker can use it, for example, to choose whether it is convenient to choose a path that is longer but with a lower slope. Additional considerations on this type of intervention follow.

##### Case Study: Intervention on Slope

From the intervention analysis results, we introduce a simple case study in which we compare two paths that reach the same destination but have a different configuration. The first path has a higher slope and a lower length, while the alternative path has a decrease in slope and therefore a higher length. By the intervention on *avg_slope*, we want to study how the fuel consumption varies and if we have a fuel saving under some configuration of the parameters intervals.

**Proof.**  Path $p_{i}$ has length $l_{i}$ and angle $\alpha_{i}$; we introduce the slope of the path as the tangent of its angle: $s_{i}=tan\alpha_{i}$.  □

**Figure 4:**
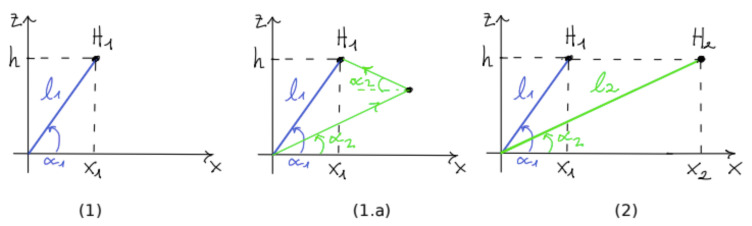
Two paths for reaching the height z=h: path (1) has a length $l_{1}$ and an angle $\alpha_{1}$; path (1.a) reaches the same destination as (1) by keeping a lower constant slope with a longer length. Path (2) is used in the proof and is equivalent to path (1.a) in the angle $\alpha_{2}$ and length $l_{2}$. The paths are compared in terms of fuel savings under different configurations, as shown in Figure 5.

We formulated the fuel consumption of path $p_{i}$ as $F_{i}=l_{i}f_{i}$, where $f_{i}$ is the *fuel_per_km* consumption related to path $p_{i}$. According to the causal effect information from Table 4, we assume that $f_{i}$ increases linearly with the slope $s_{i}=tan\alpha_{i}$. That is, from a positive slope variation $\Delta s_{i}=\Delta tan\alpha_{i}$, we have a positive $\Delta f_{i}=r\Delta \left(tan\alpha_{i}\right)$ with r=6.635. We refer to paths (1) and (2) of Figure 4, respectively, as $p_{1}$ and $p_{2}$. We observed that $p_{1}$ and $p_{2}$ are two possible paths for reaching the same destination (from Figure 4, we observe that path (2) is equivalent to path (1.a), which reaches the same destination $H_{1}$ of path (1); model (2) has a straight path to facilitate the computation of the fuel savings). We can model fuel savings as $R\left(\alpha_{1},\alpha_{2}\right)=F_{1}-F_{2}$ between path (1) and path (2). From this formulation, we ask which values of ($\alpha_{1}$,$\alpha_{2}$), with $\alpha_{2}<\alpha_{1}$, have a positive saving $R(\alpha_{1},\alpha_{2})>0$. Knowing that $f_{1}>f_{2}$, since path (1) has a greater slope than path (2), we have that $f_{1}=f_{2}+r\left(tan\alpha_{1}-tan\alpha_{2}\right)$. Therefore, we have the following:

(4) $$\begin{array}{cc} R(\alpha_{1},\alpha_{2}) & =f_{1}l_{1}-f_{2}l_{2}\\ \; & \overset{0}{=}f_{1}l_{1}-\left[f_{1}-r\left(tan\alpha_{1}-tan\alpha_{2}\right)\right]l_{2}\\ \; & \overset{1}{=}l_{2}r\left(tan\alpha_{1}-tan\alpha_{2}\right)-f_{1}\left(l_{2}-l_{1}\right)\\ \; & \overset{2}{=}rh\left(tan\alpha_{1}-tan\alpha_{2}\right)/sin\alpha_{2}-f_{1}h\left(1/sin\alpha_{2}-1/sin\alpha_{1}\right) \end{array}$$

Passage 2 of Equation (4) is found according to $l_{i}=h/sin\alpha_{i}$. We computed fuel saving $R(\alpha_{1},\alpha_{2})$ by setting the following parameters:

- $\alpha_{1}=5^{\circ }$: the angle for initial path $p_{1}$, which approximately has a 10% inclination, denotes a very steep path ($5^{\circ }$ is standardized as the maximum slope allowed for roads).
- h=0.01 km: a height of 10 m is reached by paths $p_{1}$ and $p_{2}$. We have that, for angle $\alpha_{1}$, the path $p_{1}$ has a length of about 100 m.
- $f_{1}\in \left[0.2,0.5,0.8\right]\;L/\mathrm{km}$: *fuel_per_km* consumption values for path $p_{1}$: we select them according to Table 1.
- $\alpha_{2}<\alpha_{1}$: we investigate fuel saving for paths with a lower inclination and consequently a higher length.

**Figure 5:**
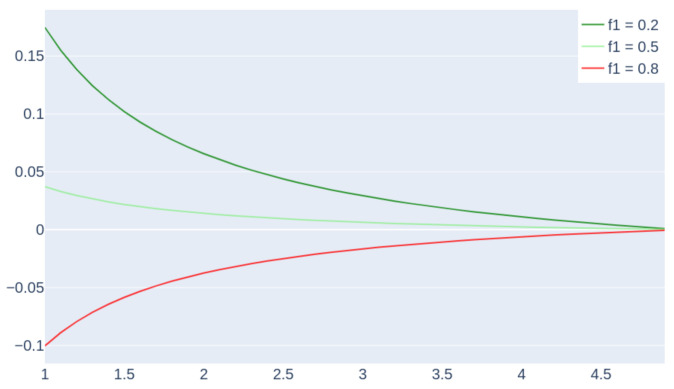
Fuel consumption saving (l) vs. slope angle $\alpha_{2}$${(}^{\circ })$ of path (2). The saving is computed by the difference in the consumption of path (1) (with fixed $\alpha_{1}=5^{\circ }$) to path (2) of Figure 4. The curves in the colors are for different values of $f_{1}\in \left[0.2,0.5,0.8\right]$ (L/km) (*fuel_per_km* consumption in path $p_{1}$).

From Figure 5, we observe that we have a positive fuel saving for $f_{1}=0.2\;L/\mathrm{km}$ and $f_{1}=0.5\;L/\mathrm{km}$, while for $f_{1}=0.8\;L/\mathrm{km}$, we waste fuel. By remembering that path $p_{2}$ has a lower angle $\alpha_{2}$ but a greater length, from the result, we see that, for greater values of $f_{1}$, we do not save fuel because the length of the path has a higher impact on the consumption. In contrast, for lower values of $f_{1}$ (starting from a threshold that is approximately 0.55 L/km), we have a positive saving because the decrease in slope has a higher impact on fuel consumption.

### 3.2. Bayes Network Validation

We compare the Bayes networks discovered with the results of the Granger experiments described in Section 2.4 on a set of multivariate time series constructed from the original data set.

Our data set is temporally confined between January and June 2019, and for each month, we generally have five consecutive dates of measurement. We exploit the temporal order of the data set and frame it as a multivariate time series collection. The path records contain information concerning the bus service: DAT (date and time of measurement) and VehicleID (identifier of the vehicle). We subset the data set into a collection of multivariate time series $\left\{t^{\left(l\right)};\;l=1,\ldots ,T\right\}$ according to VehicleID and the month of DAT. Each multivariate time series $t^{\left(l\right)}:=t_{vm}$ corresponds to the time-ordered collection of the path records measured for a given vehicle *v* on a given month *m* (we selected a time series with more than 20 temporal records and obtained a collection of T=125 multivariate time series). By formulating the data set as a time-series collection $\left\{t^{\left(l\right)}:l=1,\ldots ,T\right\}$, we obtained a set of boolean Granger matrices $\left\{G^{\left(l\right)}:l=1,\ldots ,T\right\}$, which represents the results of the Granger experiments.

Each Granger matrix $G^{\left(l\right)}$, computed from $t^{\left(l\right)}$ according to Equation (3), can be interpreted as the adjacency matrix of a graph $G^{\left(l\right)}$. Given a Bayes network B, we compared it with the set of Granger experiment graphs $\left\{G^{\left(l\right)}\right\}$ obtained for each time series of $\left\{t^{\left(l\right)}\right\}$. That is, we compare the Bayes adjacency matrix *B* with the set of Granger matrices $\left\{G^{\left(l\right)}\right\}$ by considering each element of the set as a ground truth.

We define a Bayes performance metric m(B,G), and from it, we define $M\left(B,\left\{G^{\left(l\right)}\right\}\right)=\frac{1}{T}\sum_{l=1}^{T}m\left(B,G^{\left(l\right)}\right)$ as the average Bayes performance over the collection $\left\{G^{\left(l\right)}\right\}$. We use $M(B,\left\{G^{\left(l\right)}\right\})$ as a mean of the comparison over our Bayes network collection.

#### Performance with Respect to Granger Experiments

We frame the adjacency matrices *B* and *G* as boolean vectors b and g so that each vector’s *i*th entry is a boolean indicator of the presence of link *i*. We compared two models, Bayes vector b and Granger vector g, which evaluate the presence for each link on a given link collection with size *L* (in our case, for V=7 variables, we have L=42 possible links). If we establish the Granger vector g as the ground truth of this binary classification task, we can evaluate the binary metrics of b with respect to ground truth g: that is, the number of links in the collection that are true positive (TP), true negative (TN), false positive (FP), and false negative (FN). Then, we can construct a set of Bayes performance metrics as shown in Table 5:

Sensitivity (*sens*) measures the ratio of true positives (TP) over the total positives of ground truth **g** (TP + FN), that is the percentage of g present links that are correctly identified by **b**. Specificity (*spec*) measures the ratio of true negatives (TN) over the total negatives of the ground truth g (TN + FP), that is the percentage of g absent links that are correctly identified by **b**. Average recall (*avg_recall*) measures the average between sensitivity (*sens*) and specificity (*spec*). Accuracy (*acc*) measures the ratio of true positives and true negatives (TP + TN) over the link collection size L = TP + TN + FP + FN, which is the percentage of links, whether absent or present for ground truth g, that are correctly identified by b.

**Figure 6:**
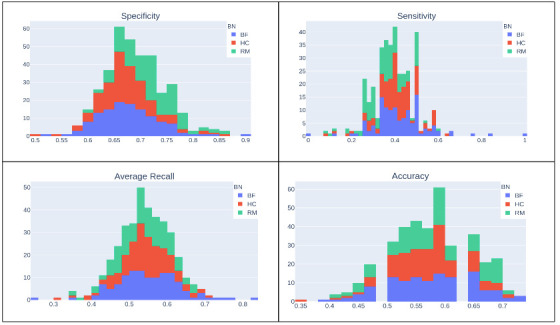
Bayes network (HC, BF, and RM) performance metric histograms (specificity on the top left, sensitivity on the top right, average recall on the bottom left, and accuracy on the bottom right) over the resulting collection of the Granger experiments performed on the multivariate time-series data set. The average of each performance metric for each Bayes network is reported in Table 6.

For each performance evaluation $m(B,G^{\left(l\right)})$ related to a given time series $t^{\left(l\right)}$ (m: sens, spec, avg_recall, and acc), we compute the averaged Bayes performance metrics $M\left(B,\left\{G^{\left(l\right)}\right\}\right)=\frac{1}{T}\sum_{l=1}^{T}m\left(B,G^{\left(l\right)}\right)$ over the time series collection $\left\{t^{\left(l\right)}\right\}$ (M: SENS, SPEC, AVG_RECALL, and ACC) for each discovered Bayes Network. We report the average performances in Table 6, where the error is computed as the standard deviation of performance over the Granger matrix collection $\left\{G^{\left(l\right)}\right\}$. Figure 6 shows the distribution of these performance metrics in the time series, for the various Bayes networks.

From Table 6, we observe that the Bayes network performances are below 60% for all metrics except for specificity. A higher sensitivity is reached by brute force (BF) which on average identifies 42% of present links with respect to the time-series Granger graphs. A higher specificity is reached by restricted maximization (RM), which identifies 72% of absent links with respect to the time-series Granger graphs. The average recall, which is the mean between sensitivity and specificity, is reasonably similar for all the networks and is around 55%. We have the same for accuracy, which is around 58%.

From Table 6, we can state that we have a poor consistency between the discovered Bayes networks and the Granger experiments since we have that, on average, these models have a low percentage of commonly identified causal relationships (e.g., accuracy for all networks is around 58%).

We can motivate these results by the following arguments:

(1) Time-series properties
  The time series may not be correlated with time and may have a consistent random component. We can verify this with Ljung–Box test [40] with total number of lags h=20 and significance level α=0.05 for each feature of the time series of our collection $\left\{t^{\left(l\right)}\right\}$. For each feature, we report the percentage of time series for which we confirm the independence assumption: *accel*, 71%; *avg_slope*, 54%; *air_cond_ptime*, 12%; *brake_usage*, 57%; *mass*, 7%; *fuel_per_km*, 57%; and *stop_ptime*, 87%. We observe that most of the features, especially *stop_ptime* and *accel*, present a high independence frequency over the time-series collection except for *aircond_ptime* and *mass*. This result may suggest that our time-series framing may be the reason for the low consistency between Granger experiments and Bayes networks.
(2) Conceptual causality difference
  We may observe that Granger test searches for causality by identifying a past temporal dependence by means of the vector auto regression model, while Bayes networks search for a present causality between features, which are collected on the same temporal level.
  Let us take an example. We consider multivariate time-series variables (A,B) for which the Granger test provides $A\overset{\left(g\right)}{\Rightarrow }B$. We have that *A* has a predictive power in forecasting *B*, but we may not be sure about the existence of a present causal dependence between *A* and *B*, that is the type of causality identified by Bayesian networks. We further explore this issue in the following section by instantiating a synthetic data set collection that reproduces these types of *past* and *present* causality.

### 3.3. Synthetic Data Study

**Figure 7:**
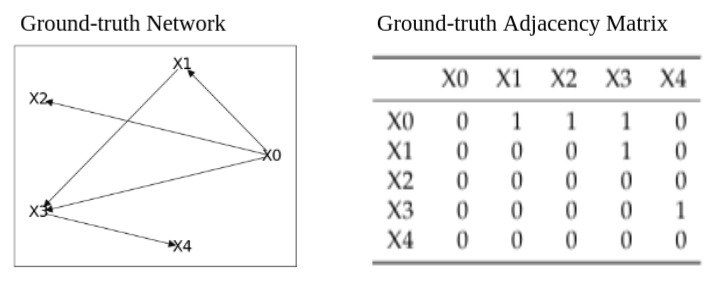
Ground truth network (**left**) and corresponding ground truth adjacency matrix (**right**). We generated a collection of synthetic data sets from the ground-truth and compared the causal inference methods of Bayes networks and Granger causality over it.

From the considerations of poor consistency between Bayes networks and Granger experiments, we decided to perform a study on a collection of synthetic data sets for which we set the causal relationship. Our synthetic data sets are publicly available at [14]. These data sets have V=5 features and N = 10,000 records $\left\{x^{\left(i\right)}=\left(x_{0}^{\left(i\right)},\ldots ,x_{4}^{\left(i\right)}\right)\;;\;i=1,\ldots ,N\right\}$, and the causal dependencies are illustrated in Figure 7:

#### 3.3.1. Synthetic Data Set Collection

We explored two types of causality: *present causality*, which is related to Bayes networks, and *past causality*, which is related to Granger causality. We explain these causalities by illustrating the corresponding data-generating processes:

- LLC: Lag linear combination
  LLC is related to *past causality*; we have that $x_{d}^{\left(t\right)}$, the *d* feature value at time step *t*, is computed as a linear combination of past lag values of all features plus an error term. In our case, let *p* be the maximum lag value; then, we have the following:
  $$x_{d}^{\left(t\right)}=\sum_{d^{\prime }=1}^{5}\sum_{l=1}^{p}a_{dd^{\prime }}^{\left(t-l\right)}\cdot x_{d^{\prime }}^{\left(t-l\right)}+\epsilon_{d}$$
  A multivariate formulation of the data generating process is as follows:
  (5) $$x^{\left(t\right)}=\sum_{l=1}^{p}A^{\left(l\right)}x^{\left(t-l\right)}+\epsilon$$
  The coefficient matrix $A^{\left(l\right)}=\left\{a_{dd^{\prime }}^{\left(t-l\right)}\right\}$ contains the linear-combination coefficients for computing $x_{d}^{\left(t\right)}$ from lag *l* feature values in each *d* row. The error vector ϵ has components distributed according to $\epsilon_{d}∼N\left(0,s\right)$.
  This data-generating process is in agreement with the *past-causality* concept and agrees with the assumptions of the vector auto regression model. Therefore, we expect highly consistent results between the ground truth and the Granger experiments.
- GCD: Gaussian conditional distribution
  GCD is related to *present causality*; we extracted $x^{\left(t\right)}$ from a multivariate joint Gaussian distribution $x^{\left(t\right)}∼p\left(x\right)$ that can be factorized in accordance with the ground truth graph as follows:
  (6) $$p\left(x\right)=\prod_{v=1}^{N}p\left(x_{v}\;|\;x_{\mathrm{pa}(v)}\right)$$
  For feature node $x_{v}$, we have that $x_{\mathrm{pa}(v)}$ is its parent set.
  This data-generating process is in agreement with the *present causality* concept since we have no dependence on past feature values as occurs in LLC; we have conditional dependencies between the feature-present values, as stated by p(x). Moreover, it is in agreement with the assumption of the algorithms for Bayes network construction (hill climbing, restrictive maximization, and brute force) for which we expect a better consistency with the ground truth.
- HYB: Hybrid data set generation
  Take two data sets generated in accordance with GCD and LLC: $\left\{x_{\left(GCD\right)}^{\left(i\right)}\right\}$ and $\left\{x_{\left(LLC\right)}^{\left(i\right)}\right\}$. We constructed an Hybrid data set modulated by parameter α∈[0,1]:
  (7) $$\left\{x_{\left(GCD\_LLC\_\alpha \right)}^{\left(i\right)}\right\}:=\left\{\alpha x_{\left(GCD\right)}^{\left(i\right)}+\left(1-\alpha \right)x_{\left(LLC\right)}^{\left(i\right)}\right\}$$
  By modulating the parameter α, we varied the weight of both *present* and *past causality* accomplished, respectively, by GCD and LLC. For low values of α, we expected a better ground truth consistency with Granger experiments, since the LLC component is more relevant. In contrast, for increasing values of α, we expect a better ground truth consistency with the Bayes network algorithms.

#### 3.3.2. Construction Parameters

We constructed five data sets in the following order:

(1) $\left\{x_{\left(LLC\right)}^{\left(i\right)}\right\}$
  For LLC generation, we used Equation (5) with only one lag value l=1. The error vector components from Equation (5) were distributed according to N(0,1). Moreover, from Table 7 and Equation (5), we observe that $X_{0}$ is a root feature of the data set since it has no variable on which it depends. We generated $X_{0}$ signal as 5sin(x/10) with the addition of noise $\epsilon_{0}∼N\left(0,1\right)$.
(2) $\left\{x_{\left(GCD\right)}^{\left(i\right)}\right\}$
  For GCD generation, we extracted the mean vector ${\bar{x}}_{\left(LLC\right)}$ and covariance matrix $\Sigma_{LLC}$ from $\left\{x_{\left(LLC\right)}^{\left(i\right)}\right\}$. In this way, we have that the statistics of GCD and LLC data sets are comparable. From the ground truth shown in Table 7, we extracted the topological order of our feature set, which is (X0,X1,X2,X3,X4). We iteratively generated each feature according to the topological order. The factors of probability distribution of Equation (6), given inital condition $x_{\mathrm{pa}(v)}=z$, are expressed as $p\left(x_{v}\;|\;x_{\mathrm{pa}(v)}=z\right)∼N\left({\tilde{\mu }}_{v},{\tilde{\sigma }}_{v}\right)$
  $$\begin{array}{cc} {\tilde{\mu }}_{v} & =\mu_{v}+s_{1,\mathrm{pa}(v)}^{T}\Sigma_{\mathrm{pa}(v),\mathrm{pa}(v)}^{-1}\left(z-\mu_{\mathrm{pa}(v)}\right)\\ {\tilde{\sigma }}_{v}^{2} & =\sigma_{v}^{2}-s_{1,\mathrm{pa}(v)}^{T}\Sigma_{\mathrm{pa}(v),\mathrm{pa}(v)}^{-1}s_{1,\mathrm{pa}(v)} \end{array}$$
  From ${\bar{x}}_{\left(LLC\right)}$ and $\Sigma_{LLC}$, we extracted the following quantities: $\mu_{\mathrm{pa}(v)}$ and $\Sigma_{\mathrm{pa}(v),\mathrm{pa}(v)}$ are, respectively, the mean vector and covariance matrix of $x_{\mathrm{pa}(v)}$; $\mu_{v}$ and $\sigma_{v}^{2}$ are, respectively, the mean and variance of $x_{v}$; and $s_{1,\mathrm{pa}(v)}$ is the covariance vector between $x_{v}$ and $x_{\mathrm{pa}(v)}$.
(3) $\left\{x_{\left(GCD\_LLC\_\alpha \right)}^{\left(i\right)}\right\};\;\left(\alpha =0.2,0.5,0.8\right)$
  We then constructed three Hybrid data sets from $\left\{x_{\left(GCD\right)}^{\left(i\right)}\right\}$ and $\left\{x_{\left(LLC\right)}^{\left(i\right)}\right\}$ by choosing α values 0.2, 0.5, and 0.8.

**Table 7:** First lag matrix for LLC synthetic data set generation from Equation (5): row $X_{m}$ contains the linear combination coefficients of the first lag values $\left(x_{0}^{\left(t-1\right)},\ldots ,x_{4}^{\left(t-1\right)}\right)$ for generating the *m* component of vector $x^{\left(t\right)}$.

	X0	X1	X2	X3	X4
X0	0	0	0	0	0
X1	1.2	0	0	0	0
X2	−1.05	0	0	0	0
X3	2.3	−1.15	0	0	0
X4	0	0	0	0.71	0

**Figure 8:**
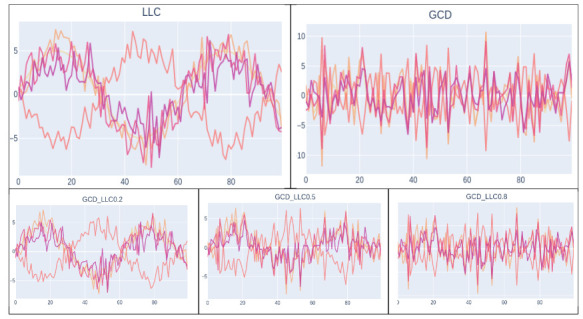
Illustrative multivariate time-series plot for each element of the synthetic data set collection. Lag linear combination (LLC) on the top left, Gaussian conditional distribution (GCD) on the top right, and hybrid (GCD_LLC_α) on the bottom for α values 0.2, 0.5, and 0.8 from left to right. We compared the Bayes network algorithms and Granger causality methods over this collection.

We display an initial portion of each synthetic data set in Figure 8. We observe that LLC features tend to have an oscillating trend since we set X0 as a sinusoidal function; GCD features are distributed in the same range of LLC, but given its data-generation process, the features do not have a correlation with time. We observe that, for GCD_LLC_α, initially, the data set displays the oscillating trend of LLC, but as α increases, it tends to be more similar to GCD.

#### 3.3.3. Results on Synthetic Data

For each synthetic data set, we evaluated the performance of the Granger causality and of Bayes networks with respect to the ground truth of Figure 7. The performance metrics are evaluated in the same way as in Table 5 with the only difference being that the ground truth is known.

We computed the Granger graph for each synthetic data set according to Equation (3) and denoted it as GC. From Section 2.4, we observe that, if we obtain a lag-value equal to 0 by fitting the VAR model with the AIC criteria, we cannot perform the Granger test (since we cannot compare auto-regressive models with 0 lags). This is the case for the GCD data set. We can observe this from Table 8, where the performance for GC is missing.

We note that, for each data set, the brute force algorithm returns a collection of $n_{BF}$ networks that we index as $BFi;i=[1,\ldots ,n_{BF}]$. This is due to the fact that brute force searches for the network with the optimal BIC score. This score can generally be obtained by more than one network. Moreover, we observe that, in most of the data sets, the discovered Bayes networks may be equal to each other. In order to avoid redundancy, we denote equal networks under the same unique name. Under this name, it groups the list of algorithms generating equal networks.

We present the collection of discovered Bayes networks for each data set:

- LLC: BF1, BF2, BF3 = HC = RM (3 networks)
- GCD: BF1, BF2 = HC = RM (2 networks)
- GCD_LLC_0.2: BF1, BF2, HC = RM (2 networks)
- GCD_LLC_0.5: BF1, BF2 = HC = RM (2 networks)
- GCD_LLC_0.8: BF1, BF2, BF3 = HC, RM (4 networks)

We note that the brute force algorithm finds two or three networks: generally, one of the BFi networks is equal to the networks obtained from HC and/or RM (except for data set GCD_LLC_0.2). The HC and RM networks are always equal except for data set GCD_LLC_0.8, where they differ for the presence of a link $X_{2}\to X_{4}$.

In Table 8, we can observe the Bayes network performances over data sets GCD and LLC. For LLC, we note that Granger causality reaches better performances with respect to the Bayes networks. This result is consistent since the data-generating process of LLC is in agreement with the VAR model under which the Granger tests are performed.

For GCD, we have an incomplete comparison since GC cannot be performed because the lag value obtained by fitting the VAR model on the GCD data set with the AIC criteria is 0. In these conditions, the Granger test cannot be performed on a VAR model with zero lag. We note that the networks (BF2 = HC = RM) perfectly discover the ground truth graph from Figure 7. This result is consistent with the data-generating process of GCD, which is in agreement with the assumptions upon which the Bayes network algorithms rely.

From Table 9, we can observe the performance of Bayes networks and Granger causality GC over the hybrid data sets GCD_LLC_α modulated by parameter α∈[0.2,0.5,0.8]. We can also note from Figure 7 that, as α increases, the contribution of GCD increases while that of LLC diminishes.

In fact, we can see that, for α=0.8, where the impact of GCD is higher, the Bayes networks (BF3 = HC and RM) obtain excellent performances in identifying the ground truth. In contrast, we note a better Granger causality (GC) performance for α=0.2, where the impact of LLC is greater.

##### Bayes Network Validation with Granger Causality

A comparison with the ground truth is feasible since we know the data-generating process. This is not the case for the real data set. In fact, in Section 3.2, we compared the Bayes networks with the collection of Granger graphs $\left\{G^{\left(l\right)}\right\}$ obtained from the Granger tests over the multivariate time-series collection $\left\{t^{\left(l\right)}\right\}$. According to this previous method, we performed a Bayes network comparison for each synthetic data set with respect to the Granger graph constructed for each data set of our synthetic collection. We note that this comparison can be performed on all data sets of the synthetic collection except for GCD.

We present the Bayes network performances by identifying the Granger graph ground truth for each synthetic data set in Table 10. First, we note that the Bayes network performances are generally lower with respect to those in Table 8 and Table 9. This may also be due to the fact that we do not validate the networks with the real ground truth of Table 7 but with the Granger causality experiments, which may not properly reflect the effective ground truth. By focusing on the average recall AVG_RECALL, we do not have a noticeable positive performance variation between the data sets, except for networks (HC = RM) for which we have 0.9 average recall on a hybrid data set with α=0.2.

##### Identification of Ground Truth Links

In order to make a more detailed study of the Bayes and Granger experiments over the synthetic data set, we perform a grouping of the results for each ground truth link from Figure 7 and for each link state in the resulting network. The link state in the result graph can be *Present* (present link with correct direction), *Inverse* (present link but with inverse direction), or *Absent* (absent link), as we can observe from Table 11.

From Table 11, we observe that, for each ground truth link, most of the resulting graphs identify the links. This is also confirmed by the satisfactory performances of Table 8 and Table 9. We have that some resulting graphs identify the link but with inverse directions, especially some of the brute force networks and, less frequently, the Granger causality and the HC and RM networks. Finally, we have that the only ground truth link found to be absent by some resulting graphs is X3→X4.

Concerning the false-positive links: they are links identified by the resulting graphs but not present in the ground truth. We have that, for LLC, all BF nets find the link X1→X4 while GC finds X4→X1. For GCD, no false-positive links are found. This means that the Bayes networks are very precise in determining the ground truth, mainly because GCD is the data-generating process for which they are more in agreement. For GCD_LLC_0.2, GC finds X0→X4 and X1→X4. For GCD_LLC_0.5, all Bayes networks find the links X1→X2 (except for BF1, which finds X2→X1) and X0→X4. For GCD_LLC_0.8, all of the resulting graphs find the links X2→X4 (except for RM). GC also finds X2→X3. We can say that this apparent causal relationship may be found because we can find a path in the ground truth graph in Figure 7 that connects the cause and the effect variables. As an example, we observe that the link X0→X4 does not exist but the that two variables have a path joining them through X1 and X3.

## 4. Conclusions

This work presents different contributions with the purpose of analyzing the conditions at which fuel consumption occurs in vehicles and of understanding how to reduce it by intervening in the scenario. We provided a collection of data from sensors installed on buses used as public transport. Thanks to the sensor data analysis, we discovered that, in some contextual conditions (with a fuel consumption per kilometer that does not exceed the value of 0.75 L per kilometer), it is preferable to choose a longer but less steep path than a shorter one. As a consequence of the analysis of cause–effect relationships between the variables and the target, we precisely quantified the impact of all causes on the target: with a decrease of one unit of air_cond_ptime (percentage of travel time with air conditioning), we can can expect a decrease of 0.107 units in fuel_per_km; with a decrease of one unit of the percentage of time with brake_usage, we can expect a decrease of 0.206 units in fuel_per_km; and with a decrease of a unit in stop_ptime (stop percentage time with engine on), we can expect a decrease of 0.445 units in fuel_per_km. In the literature [6], the important effect of this variable was confirmed.

We tested both approximate algorithms, driven by the BIC score and brute force with the purpose of comparing the ability of the algorithms to converge to the same resulting networks. We evaluated their results with the adoption of Granger causality, a third-party criterion, based on the time series formed in time by the observed variables. This is an original contribution to the scientific community of Bayesian networks that are usually scored by BIC or K2. According to the Granger causality, we are also able to rank the alternatives, even in the case where multiple BNs share the same score. We compared BNs also by using their ability to perform feature selection and to predict the target variable.

We also provided a synthetic data set that we created with a known ground truth of which the purpose is to test the algorithms of synthesis of BN from data and to verify their convergence toward the ground truth. We discussed the comparison results. The networks sometimes agree, and other times, they do not. This mismatch perhaps is due to the multiple maxima that sometimes exist in the large search space of the solutions: this occurs especially in the synthetic data in which the ground truth is known and in which the data determine similar links between cause and effect, but in opposite directions. The observed mismatches on the edges might also be a consequence of the heuristics. Heuristics are indeed used to eliminate multiple rankings of the alternatives, in choosing edge directions (choice of the cause and the effect that often requires the experts’ advises), and for avoiding cycles in the BN graphs.

In summary, the contributions of our work are as follows:

1. Bayesian networks were applied for the analysis of fuel consumption. Past studies on fuel consumption in vehicles (reported in Section 1) applied only machine learning predictive models (based on SVR, ANN, random forest, or gradient boosting). All of them have the sole goal of predicting the target value. None provide machine learning models that are able to also perform the following:
  (a) describing and discovering the cause–effect relationships between variables and the target (Section 3) and
  (b) performing an intervention analysis on the causes, with the goal of achieving a desired impact on the target and quantifying this impact (Section 3.1.3).
  Bayesian networks are powerful and we used them to reach multiple goals: perform feature selection (Section 3.1.2) whose outcomes we compared with another standard method (VIF [37]); perform predictive modeling (target estimation, whose results are shown in Table 3), scenario simulation (Section 3.3.1), intervention analysis (Section 3.1.3) and counterfactual analysis (what-if analysis).
2. Comparing the results of approximate algorithms (heuristic-driven) for Bayesian networks with a brute force algorithm, an original one, implemented for this work (Algorithm 1) was made possible thanks to the availability of high-performance computing technology that permits us to afford an extremely high computational load of traversing the huge search space of the possible networks by partitioning it and spreading evaluations of the alternative graphs throughout many servers. The outcome of this comparison (Section 3.2) can help analysts with the uncertainty of which Bayesian network to use.
3. The use of the Granger causality concept was introduced and formalized for an evaluation of Bayesian networks (Section 2.4). Granger causality was used as an independent, third party notion to compare, evaluate, and rank the different Bayesian networks, generated from the same data by different algorithms.
4. Bayesian network discovery is customarily used to test the domain knowledge, previously distilled under the form of an already available graph [22,23,36,41]. Differently, in this paper, we did not start from an already available graph but directly started from the collected (sensor) data and provided experts with assumptions about this knowledge (cause–effect relationships) under the form of a Bayesian network.
5. Last but not least, we provided two public data sets to the scientific community [14] with real data from buses and a synthetic data set with ground truths, useful for testing Bayesian network algorithms and time series analyses.

## Figures and Tables

**Table 1:** Statistics of the data set features: mean, std (standard deviation), and minimum and maximum feature values.

	Mean	Std	Min	Max
avg_slope (%)	0.00	0.02	−0.30	0.21
mass (ton)	21.19	1.55	17.92	29.85
aircond_ptime (%)	0.0	0.2	0.0	1.0
stop_ptime (%)	0.19	0.15	0.01	0.97
brake_usage (%)	0.20	0.09	−0.06	0.72
accel (m/s^2^)	0.36	0.21	0.01	1.80
fuel_per_km (L/km)	0.57	0.20	0.02	3.93

**Table 3:** Ten-fold cross validation (CV)-averaged *rmse* score for each regression of *target* (column) on possible parent sets identified by a Bayes network collection: {brute force (BF), hill climbing (HC), or restrictive maximization (RM)}. The first three rows list the possible parent set and ordered CV scores together with the Bayes networks that generate the given parent set. Detailed information on the parent set can be retrieved from Figure 3. The last two rows display the *target* mean and standard deviation.

	Accel	fuel_per_km	brake_usage	stop_ptime	aircond_ptime	Mass
CV1	0.18 (RM)	0.13 (HC, BF)	0.07 (BF)	0.15 (HC)	0.13 (HC, BF, RM)	1.6 (BF)
CV2	0.18 (HC)	0.14 (RM)	0.08 (HC, RM)			
CV3	0.21 (BF)					
std	0.21	0.20	0.09	0.15	0.18	1.6
mean	0.37	0.58	0.20	0.19	0.04	21.2

**Table 4:** Causal effects of variables on target variable fuel_per_km.

Variable	Adjustment Set	Causal Effect
slope	{ }	6.635
mass	{slope,brake_usage,accel}	0.012
air_cond_ptime	{mass}	0.107
stop_ptime	{ }	0.445
brake_usage	{slope,stop_ptime,accel}	0.206
accel	{slope,stop_ptime}	0.189

**Table 5:** Binary metrics of Bayes vector b with respect to ground truth g (b and g are boolean link-indicator vectors of a link collection of size *L*). From binary metrics, Bayes performance metrics are evaluated: sensitivity (*sens*), specificity (*spec*), average recall (*avg_recall*), and accuracy (*acc*).

Binary Metrics	Bayes Network Performance Metrics
$TP=\sum_{i=1}^{L}I\left(b_{i}=1,g_{i}=1\right)$	sens=TP/(TP+FN)
$TN=\sum_{i=1}^{L}I\left(b_{i}=0,g_{i}=0\right)$	spec=TN/(TN+FP)
$FP=\sum_{i=1}^{L}I\left(b_{i}=1,g_{i}=0\right)$	avg_recall=(sens+spec)/2
$FN=\sum_{i=1}^{L}I\left(b_{i}=0,g_{i}=1\right)$	acc=(TP+TN)/(TP+TN+FP+FN)

**Table 6:** Bayes average performance metrics over the Granger experiment set $\left\{G^{\left(l\right)}\right\}$: sensitivity (SENS), specificity (SPEC), average recall (AVG_RECALL), and accuracy (ACC) evaluated for the Bayes networks (HC, BF, and RM). The metrics represent the level of accordance between the Bayes causality models and the collection of results obtained by the Granger experiments. In bold the best results.

	SENS	SPEC	AVG_RECALL	ACC
BF	**0.42** ± **0.12**	0.68 ± 0.06	**0.55** ± **0.09**	**0.58** ± **0.08**
HC	0.41 ± 0.09	0.68 ± 0.05	0.54 ± 0.07	0.57 ± 0.06
RM	0.36 ± 0.09	**0.72** ± **0.05**	0.54 ± 0.07	**0.58** ± **0.07**

**Table 8:** Granger causality (GC) and Bayes networks (brute force networks (BFi) and hill climbing (HC)) performances with respect to the synthetic (Table 7) data sets LLC and GCD.

	LLC				GCD	
	**BF1**	**BF2**	**BF3 = HC = RM**	**GC**	**BF1**	**BF2 = HC = RM**
SENS	0.40	0.60	0.80	1.00	0.60	1.0
SPEC	0.75	0.80	0.85	0.90	0.90	1.0
ACC	0.68	0.76	0.84	0.92	0.84	1.0
AVG_RECALL	0.57	0.70	0.82	0.95	0.75	1.0

**Table 9:** Granger causality (GC) and Bayes networks (brute force networks (BFi) and hill climbing (HC)) performances with respect to the synthetic ground truth (Table 7) for data sets GCD_LLC_α for α=(0.2,0.5,0.8).

	α=0.2				α=0.5			α=0.8				
	**BF1**	**BF2**	**HC = RM**	**GC**	**BF1**	**BF2 = HC = RM**	**GC**	**BF1**	**BF2**	**BF3 = HC**	**RM**	**GC**
SENS	0.60	0.60	0.80	1.00	0.80	0.80	1.00	0.80	0.60	1.00	1.0	0.80
SPEC	0.80	0.80	0.85	0.90	0.85	0.85	0.50	0.90	0.85	0.95	1.0	0.75
ACC	0.76	0.76	0.84	0.92	0.84	0.84	0.60	0.88	0.80	0.96	1.0	0.76
AVG_RECALL	0.70	0.70	0.82	0.95	0.82	0.82	0.75	0.85	0.72	0.98	1.0	0.78

**Table 10:** Bayes network (brute force networks (BFi) and hill climbing (HC)) performances with respect to Granger graph ground truth computed rfor each synthetic data set: LLC and GCD_LLC_α for α=(0.2,0.5,0.8).

	LLC			α=0.2			α=0.5		α=0.8			
	**BF1**	**BF2**	**BF3 = HC = RM**	**BF1**	**BF2**	**HC = RM**	**BF1**	**BF2 = HC = RM**	**BF1**	**BF2**	**BF3 = HC**	**RM**
SENS	0.43	0.43	0.57	0.43	0.57	0.86	0.40	0.47	0.44	0.33	0.44	0.44
SPEC	0.78	0.78	0.83	0.78	0.83	0.94	0.90	1.00	0.88	0.81	0.88	0.94
ACC	0.68	0.68	0.76	0.68	0.76	0.92	0.60	0.68	0.72	0.64	0.72	0.76
AVG_RECALL	0.60	0.60	0.70	0.60	0.70	0.90	0.65	0.73	0.66	0.57	0.66	0.69

**Table 11:** Grouping of causality graphs obtained from the Bayes networks and Granger experiments (GC) for each synthetic data set. We compared the graph links with the ground truth links (columns) in Figure 7. The link can be *Present* (present link in the graph and ground truth), *Inverse* (present link with an inverse direction), *Absent* (absent link in the graph and present in ground truth).

		Ground Truth Links				
	**Data Set**	**X0 → X1**	**X0 → X2**	**X0 → X3**	**X1 → X3**	**X3 → X4**
Present Link	LLC	BF3 = HC = RM; GC	BF2; BF3 = HC = RM; GC	BF1; BF2; BF3 = HC = RM; GC	BF1; BF2; BF3 = HC = RM; GC	GC
	GCD	BF2 = HC = RM	BF1; BF2 = HC = RM	BF2 = HC = RM	BF1; BF2 = HC = RM	BF1; BF2 = HC = RM
	GCD_LLC0.2	BF1; BF2; HC = RM; GC	BF1; BF2; HC = RM; GC	BF1; BF2; HC = RM; GC	HC = RM; GC	GC
	GCD_LLC0.5	BF1; BF2 = HC = RM; GC	BF1; BF2 = HC = RM; GC	BF1; BF2 = HC = RM; GC	BF1; BF2 = HC = RM; GC	GC
	GCD_LLC0.8	BF1; BF3 = HC; RM; GC	BF2; BF3 = HC; RM; GC	BF1; BF3 = HC; RM; GC	BF1; BF2; BF3 = HC; RM; GC	BF1; BF2; BF3 = HC; RM
Inverse Link	LLC	BF1; BF2	BF1			
	GCD	BF1		BF1		
	GCD_LLC0.2				BF1; BF2	
	GCD_LLC0.5	GC				BF1; BF2 = HC = RM; GC
	GCD_LLC0.8	BF2; GC	BF1; GC	BF2		
Absent Link	LLC					BF1; BF2; BF3 = HC = RM
	GCD					
	GCD_LLC0.2					BF1; BF2; HC = RM
	GCD_LLC0.5					
	GCD_LLC0.8					GC

## Data Availability

Data are publicly available at: https://github.com/rosameo/Sensors-Data-about-Fuel-Consumption-in-Buses (accessed on 8 July 2021).
